# Efficacy and safety of baihe gujin decoction as an adjunct to chemotherapy in pulmonary tuberculosis: A systematic review and meta-analysis

**DOI:** 10.3389/fphar.2025.1538692

**Published:** 2025-05-13

**Authors:** Yilu Zhao, Yiran Han, Jia Liu, Honghong Niu, Peilong Wang, Yuxi Li, Jianqin Liang, Wenping Gong

**Affiliations:** ^1^ Institute of Tuberculosis, Senior Department of Tuberculosis, The Eighth Medical Center of PLA General Hospital, Beijing, China; ^2^ Hebei North University, Zhangjiakou, Hebei, China; ^3^ Department of Infectious Disease, Beijing Geriatric Hospital, Beijing, China

**Keywords:** pulmonary tuberculosis, Baihe Gujin decoction, anti-tuberculosis drugs, meta-analysis, randomized controlled trial

## Abstract

**Background:**

Pulmonary tuberculosis (PTB), an ancient affliction, continues to present significant challenges in modern medicine. Baihe Gujin Decoction, a traditional Chinese botanical drug remedy, has been widely utilized in clinical practice for tuberculosis treatment, yet its efficacy has been inconsistent. This meta-analysis aims to ascertain its effectiveness and contribute to evidence-based medicine.

**Methods:**

A comprehensive search was conducted across multiple databases, including PubMed, Embase, The Cochrane Library, China Science and Technology Journal Database, Wanfang Database, China Biomedical Literature Database, and China National Knowledge Infrastructure, to identify relevant randomized controlled trials from January 2010 to February 2024. The risk of bias in the included studies was assessed using the Cochrane Collaboration’s tool, and meta-analyses were performed using Review Manager and Stata to evaluate the comparative outcomes.

**Results:**

This meta-analysis encompassed 32 studies. The control group exhibited a notably higher clinical overall efficacy rate [OR = 5.50, 95%CI (4.18, 7.24), *P* < 0.05], lesion absorption rate [OR = 5.83, 95%CI (4.08, 8.33), *P* < 0.05], cavity change rate [OR = 2.35, 95%CI (1.50, 3.69), *P* < 0.05], and sputum negative conversion rate [OR = 2.85, 95%CI (2.12, 3.83), *P* < 0.05]. In contrast, the treatment group demonstrated an increase in CD4^+^ T lymphocyte subset levels post-treatment, with a weighted mean difference of [OR = 4.87, (95%CI (1.91, 7.83), *P* < 0.05]. Furthermore, safety indices, including the incidence of total adverse reactions, liver function abnormalities, and gastrointestinal reactions, were significantly lower in the treatment group.

**Conclusion:**

The combination of Baihe Gujin Decoction with biomedicine is more efficacious than biomedicine alone for treating PTB. This superiority is evident in improved clinical efficacy rates, lesion absorption, cavity changes, sputum negative conversion rates, and immune indices, alongside a reduced incidence of adverse reactions.

**Systematic review registration:**

https://www.crd.york.ac.uk/PROSPERO/, CRD42023462056

## Graphical Abstract

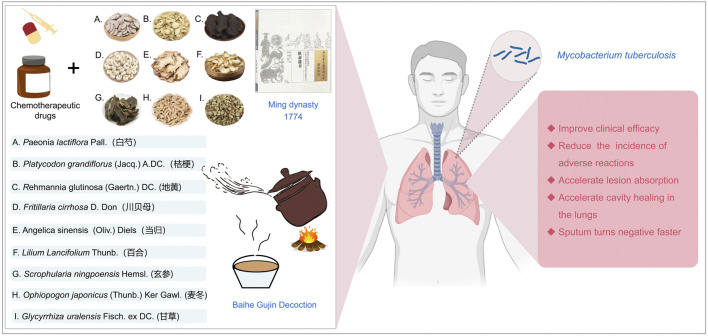

## 1 Introduction

Pulmonary tuberculosis (PTB), characterized by chronic infection of the lungs, remains a formidable threat to global health. Identified as the causative agent, *Mycobacterium tuberculosis* (MTB) has emerged as a leading cause of mortality from infectious diseases, second only to COVID-19 (Peng et al., 2024). In 2023 alone, tuberculosis (TB) claimed an estimated 1.08 million lives among HIV-negative individuals (WHO, 2024; Chen et al., 2025). Despite advancements in TB management through strategic treatment protocols and anti-tuberculosis drugs such as isoniazid, rifampicin, pyrazinamide, and either ethambutol or streptomycin, the prevalence of adverse drug reactions often leads to treatment discontinuation, hindering efforts to control the disease. The conventional chemotherapy for PTB, while effective, necessitates prolonged treatment durations and is not without its drawbacks, including a high incidence of adverse reactions that can compromise patient adherence and therapeutic success. This underscores an urgent need for innovative pharmacotherapies that are both safe and efficacious, enhancing patient compliance and bolstering the global fight against TB.

Traditional Chinese Medicine (TCM), deeply rooted in China’s cultural heritage, has made significant contributions to the prevention and management of TB within the country (Liang et al., 2021). From a TCM perspective, PTB is classified as “Fei Lao,” attributed to a deficiency in vital energy and the invasion of pathogenic factors into the lungs (Chen, 2020; Su HT et al., 2020). TCM, with its extensive array of natural metabolites, coherent theoretical framework, and minimal side effects, offers distinct advantages in managing PTB. It employs strategies that aim to strengthen the body’s essential energy, expel pathogens, nourish the liver and kidneys, enhance blood circulation, and address both the root causes and specific manifestations of the disease. As a complementary therapy, TCM not only boosts immune responses but also accelerates lesion resolution and aids in swift recovery. The integration of TCM with Chemotherapy has the potential to reduce adverse reaction rates, improve patient compliance, and achieve more favorable therapeutic outcomes (Lei FY and Wang, 2006). This approach highlights the value of combining traditional and modern medical practices for a holistic treatment of PTB.

**FIGURE 1:**
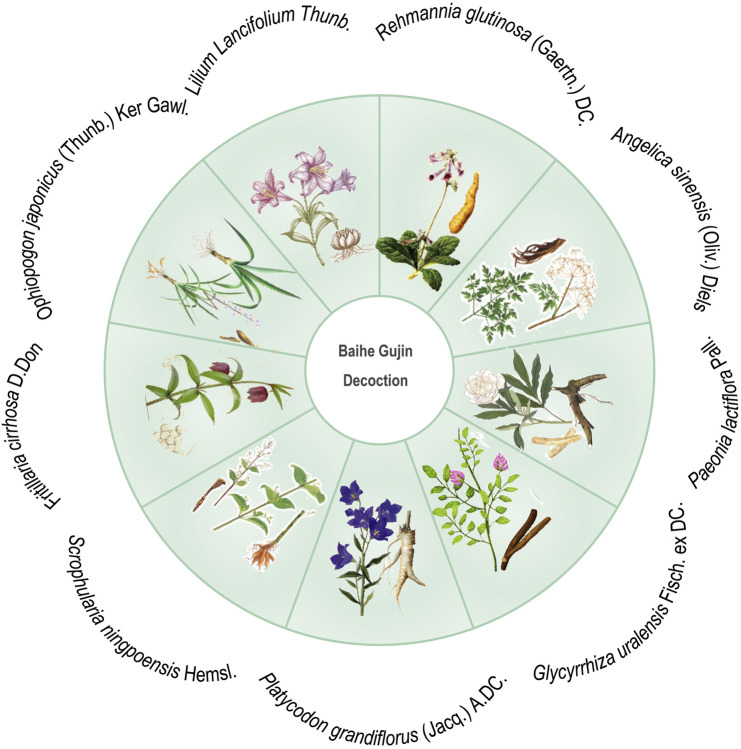
Schematic diagram of botanical drugs involved in Baihe Gujin Decoction formula.

Baihe Gujin Decoction (BGD), a TCM prescription formula, is composed of nine botanical drugs known for their potent immunomodulatory, including *Lilium Lancifolium* Thunb. [Liliaceae; Lilii bulbus], *Rehmannia glutinosa* (Gaertn.) DC. [Scrophulariaceae; Rehmanniae radix], *Angelica sinensis* (Oliv.) Diels [Apiaceae; Angelicae sinensis radix], *Paeonia lactiflora* Pall. [Paeoniaceae; Paeoniae radix], *Glycyrrhiza uralensis* Fisch. ex DC. [Fabaceae; Glycyrrhizae radix et rhizoma], *Platycodon grandiflorus* (Jacq.) A.DC. [Campanulaceae; Platycodonis radix], *Scrophularia ningpoensis* Hemsl. [Scrophulariaceae; Scrophulariae radix], *Fritillaria cirrhosa* D. Don [Liliaceae; Fritillariae cirrhosae bulbus] and *Ophiopogon japonicus* (Thunb.) Ker Gawl. [Asparagaceae; Ophiopogonis radix] (Figure 1). In attention to anti-inflammatory, antioxidant, and antimicrobial properties, it exhibits spasmolytic, anti-asthmatic, and expectorant effects, making it particularly effective in treating conditions such as PTB, chronic bronchitis, hemoptysis secondary to bronchiectasis, chronic pharyngitis, and spontaneous pneumothorax (Ge and Zhu, 2020; Jia et al., 1992). The clinical efficacy of BGD in PTB patients has been supported by various systematic reviews (Liu et al., 2022). However, its antimicrobial action against MTB and other pathogens is relatively moderate, suggesting that its therapeutic potential may be attributed to its regulatory effects on immune function (Wang et al., 2022). In managing MTB infection, combining BGD with conventional anti-tuberculosis pharmacotherapy is advisable to optimize patient outcomes. As we all know, one of the most common side effects of biomedicine is a strong liver damage. Wang et al. found that the combination of BGD and modern anti-TB drugs in the treatment of TB is not only beneficial to the improvement of symptoms and recovery of health, but also reduces the liver damage caused by anti-TB drugs (Wang, 2004). BGD is clearly preferred over other TCM formulas because it has a longer history of treating TB and is one of the preferred formulas for treating TB in combination with biomedicine. Despite numerous studies on the drug composition, metabolites, clinical efficacy, and mechanism of action of BGD, there is a paucity of specific evaluations regarding the combination therapy of BGD with anti-tuberculosis drugs for PTB.

To bridge this knowledge gap, the present systematic meta-analysis incorporates randomized controlled trials (RCTs) to compare the efficacy and safety of BGD combined with anti-tuberculosis drugs *versus* the use of anti-tuberculosis drugs alone for PTB treatment. This study aims to provide a more comprehensive evaluation of the effectiveness of BGD in combination therapy, offering more reliable evidence for clinical practice.

## 2 Materials and methods

This study strictly adhered to the guidelines set forth by the Preferred Reporting Items for Systematic Reviews and Meta-Analyses (PRISMA) statement. A PRISMA checklist is provided as a supplementary file (Supplementary File S1), and the protocol for this review has been registered with the International Prospective Register of Systematic Reviews (PROSPERO: CRD42023462056).

### 2.1 Literature search strategy

A thorough computer-based search was conducted across both Chinese and English databases to ensure a comprehensive literature review. The search timeframe from database inception to 1 September 2024. The databases included PubMed, Embase, The Cochrane Library, Wanfang Database, China National Knowledge Infrastructure, The China Science and Technology Journal Database, and China Biomedical Literature Database. The search strategy was meticulously crafted to encompass relevant studies, utilizing terms such as “Baihe Gujin Decoction,” “pulmonary tuberculosis,” “Traditional Chinese Medicine,” and “tuberculosis”. The dual-language approach aimed to ensure the inclusion of all pertinent studies, as detailed in Table 1.

**TABLE 1:** Search strategy.

Databases	Search strategy
PubMed	((Pulmonary Tuberculosis [MeSH Major Topic]) OR ((Tuberculoses, Pulmonary):ab,ti,kw OR (Pulmonary Tuberculoses):ab,ti,kw OR (Pulmonary Tuberculosis):ab,ti,kw OR (Pulmonary Consumption):ab,ti,kw OR (Consumption, Pulmonary):ab,ti,kw OR (Consumptions, Pulmonary):ab,ti,kw OR (Pulmonary Consumptions):ab,ti,kw OR (Pulmonary Phthisis):ab,ti,kw OR (Phthises, Pulmonary):ab,ti,kw OR (Phthisis, Pulmonary):ab,ti,kw OR (Pulmonary Phthises):ab,ti,kw [Title/Abstract])) AND (Baihe Gujin Decoction [Title/Abstract]) OR (Traditional Chinese Medicine):MeSH])
Embase	#1 = ‘lung tuberculosis’/exp
	#2 = ‘lung tuberculosis’:ab,ti OR ‘lung tb’:ab,ti OR ‘pulmonary tb’:ab,ti OR ‘lung tuberculous cavity’:ab,ti OR ‘chronic pulmonary tuberculosis’:ab,ti
	#3 = #1 OR #2
	#4 = ‘baihe gujin decoction’ OR ‘baihe gu jin decoction’ OR ‘Traditional Chinese Medicine’
	#5 = #3 And #4
The Cochrane Library	#1 Tuberculosis, Pulmonary
	#2 (Tuberculoses, Pulmonary):ab,ti,kw OR (Pulmonary Tuberculoses):ab,ti,kw OR (Pulmonary Tuberculosis):ab,ti,kw OR (Pulmonary Consumption):ab,ti,kw OR (Consumption, Pulmonary):ab,ti,kw OR (Consumptions, Pulmonary):ab,ti,kw OR (Pulmonary Consumptions):ab,ti,kw OR (Pulmonary Phthisis):ab,ti,kw OR (Phthises, Pulmonary):ab,ti,kw OR (Phthisis, Pulmonary):ab,ti,kw OR (Pulmonary Phthises):ab,ti,kw
	#3 = #1 OR #2
	#4 (Baihe Gujin decoction):ti,ab,kw OR (Baihe Gujin*):ti,ab,kw OR (Traditional Chinese Medicine):ti,ab,kw
	#5 = #3 AND #4
Wanfang Database	主题: (百合固金汤or百合固金汤加味or百合固金汤加减) and 主题: (肺结核)
China National Knowledge Infrastructure	主题: (百合固金汤+百合固金汤加味+百合固金汤加减) AND主题: (肺结核 + 肺结核病 + 典型肺结核)
The China Science and Technology Journal Database	题名或关键词 = 百合固金汤or百合固金汤加味or百合固金汤加减 AND 题名或关键词 = 肺结核
Chian Biomedical Literature	(“百合固金汤” [常用字段:智能] OR “百合固金汤加味” [常用字段:智能] OR “百合固金汤加减” [常用字段:智能] AND “肺结核” [常用字段:智能])

Furthermore, a reference search methodology was employed, which involved scrutinizing the reference lists of identified articles to uncover additional relevant studies. This rigorous search strategy was designed to enhance the robustness and reliability of our meta-analysis.

### 2.2 Inclusion and exclusion criteria

The inclusion criteria were as follows: (1) study subjects diagnosed with PTB based on clinical diagnostic criteria, irrespective of age or gender; (2) intervention measures where the control group received conventional anti-TB treatment with chemical drugs alone, and the treatment group received BGD in conjunction with conventional anti-TB treatment; (3) language restrictions limited to Chinese or English literature.

The exclusion criteria included: (1) duplicated publications; (2) literature with incomplete or incorrect data, or lacking complete content; (3) non-randomized controlled trials; (4) case reports, conference records, and review articles.

### 2.3 Data extraction and quality assessment

The included literature was subjected to risk assessment using the Cochrane Risk of Bias Assessment Tool. Two independent researchers performed literature screening and data extraction, with disagreements resolved through consultation with the corresponding author. The primary outcome measures extracted included the clinical efficacy rate and lesion absorption rate. Secondary outcome measures encompassed sputum negative conversion rate, changes in cavities, the incidence of adverse reactions, such as liver function abnormalities, gastrointestinal reactions, and CD4^+^ T lymphocyte subset levels post-treatment.

### 2.4 Statistical analysis

The meta-analysis data were rigorously analyzed using RevMan (version 5.4; Copenhagen, Denmark) and Stata (version SE 15.1; College station, TX, United States of America), employing a dual-software approach to ensure robustness and accuracy (Wang et al., 2023). Dichotomous variables were expressed as odds ratios (ORs) with 95% confidence intervals (CIs), assessing the strength of association between interventions and outcomes. Continuous variables were articulated as weighted mean differences (WMDs) with 95% CIs, facilitating comparison of average outcomes between groups.

Heterogeneity among the included studies was evaluated, with significant heterogeneity indicated by P-values ≤0.05 or I^2^ ≥ 50%. A random-effects model was selected for analysis in such cases, acknowledging variability across studies. A fixed-effects model was applied when P-values >0.05 and I^2^ < 50%, assuming observed variability was due to chance.

Publication bias was assessed using a funnel plot and Egger’s quantitative test, crucial for evaluating potential bias. Sensitivity analysis was conducted using Stata 15.1 by sequentially excluding leading outcome measure indicators. The stability and reliability of the results were inferred from the absence of significant changes upon these exclusions. The significance level for the meta-analysis was set at an alpha (α) of 0.05, establishing a rigorous threshold for statistical significance.

## 3 Results

### 3.1 Literature screening process

**FIGURE 2:**
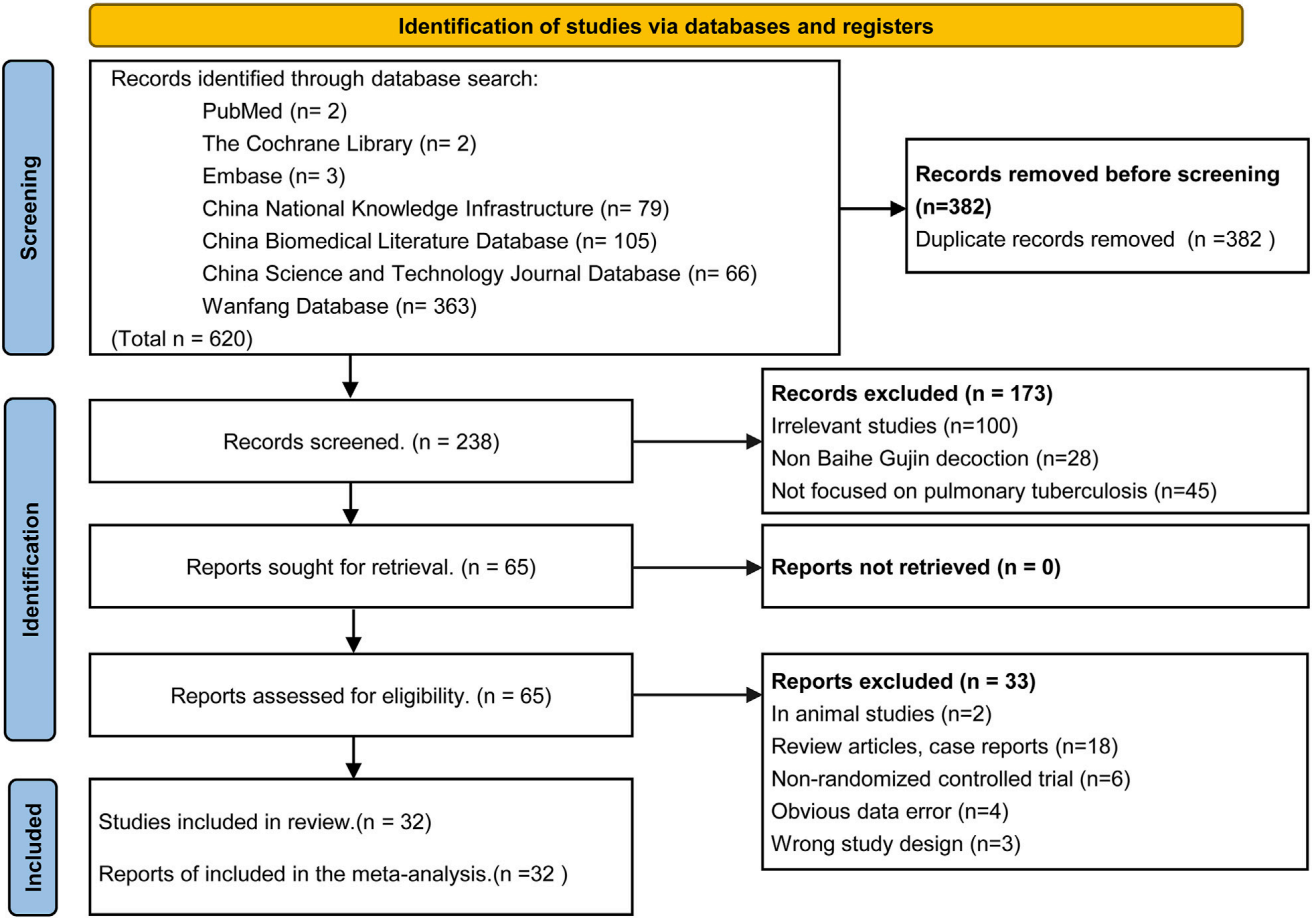
Flow diagram of the article selection process (PRISMA 2020).

**FIGURE 3:**
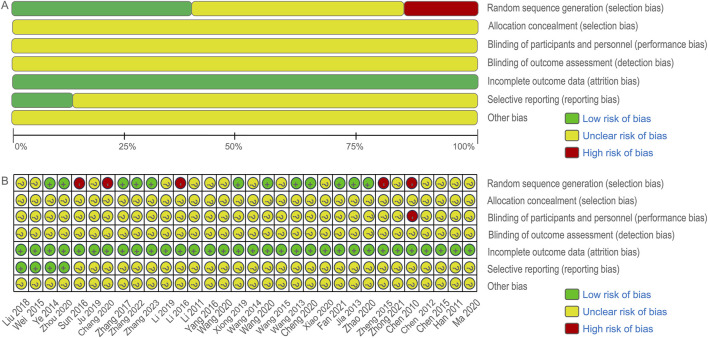
Cochrane risk bias assessment tool.

The initial search yielded 620 articles, of which 238 remained after duplicate removal using Endnote X9 software. Following title and abstract review, 173 articles were excluded, leaving 65 articles for initial inclusion. Full-text screening led to the exclusion of 33 articles for various reasons, including two animal experiments, four articles with data errors, six non-randomized controlled trials, 18 case reports and reviews, and three for other reasons. Ultimately, 32 RCTs were included in the analysis, of which only 27 explicitly mentioned random allocation concealment and 12 were double-blind designs. The literature screening process is depicted in Figure 2, and the risk of bias in the included studies is presented in Figure 3.

### 3.2 Characteristics of included studies

Table 2 summarizes the main characteristics of the included studies, which were published between 2010 and 2024 and originated from Chinese publications. A total of 2,761 patients were randomly assigned to treatment with either anti-tuberculosis drugs alone or a combination therapy with BGD, as detailed in the Jadad scale provided in Supplementary File S2.

**TABLE 2:** Characteristics of the included studies.

Study ID	Sample size (C/T)	Male/Female (C/T)	Mean age (years, C/T)	Control group	Treatment group	Durations (days)	Outcomes	JADAD
Li 2016 (Li, 2016)	46/49	23:23/28:21	48.0 ± 2.9/49.0 ± 3.6	National standard chemotherapy regimen	BGD + C	30d	①	4
Jia 2013 (Jia, 2013)	59/59	41:18/39:40	49 ± 4.89/51 ± 5.57	National standard chemotherapy regimen	BGD + C	7d is one course of treatment, with 1 month of treatment	①	3
Liu 2018 (Liu, 2018)	54/54	23:41/24:30	44.29 ± 6.24/42.13 ± 5.88	6ZAmLfxPASPto (Cs)/18ZLfxPtoPAs scheme	BGD + C	180d	①③⑧	4
Xiao 2020 (Xiao, 2020)	82/82	42:40/44:38	50.97 ± 3.48/51.02 ± 3.53	HRZES (Qod) scheme	BGD + C	7d is one course of treatment, with 1 month of treatment	①	2
Chen 2015 (Chen et al., 2015)	30/30	18:12/16:14	36.7 ± 1/40.2 ± 1	2HRZE/4HR scheme	BGD + C	60d	①②⑤⑥⑦	3
Wei 2015 (Wei, 2015)	34/34	NA	NA	2HRZE/4HR scheme	BGD + C	180d	①⑤⑦	4
Zheng 2015 (Zheng, 2015)	60/60	NA	NA	6ZKm (Am,Cm)Lfx (Mfx)Cs(PAS,E)Pto/18ZLfx (Mfx)Cs(PAS,E)Pto scheme	BGD + C	180d	①②③	4
Chen 2010 (Chen and Zhou, 2010)	35/40	NA	NA	National standard chemotherapy regimen	BGD + C	180d	①②③④	2
Ye 2014 (Ye, 2014)	37/38	24:13/26:12	39/37	2HRZE/4HR scheme	BGD + C	180d	②③	5
Li 2011 (Li, 2011)	30/30	18:12/20:10	NA	3PaAmThVZ/15PaThVZ scheme	BGD + C	90d	②	4
Chang 2020 (Chang and Jin, 2020)	75/75	48:27/45:30	47.53 ± 3.68/45.37 ± 3.41	2HRZE/4HR scheme	BGD + C	90d	①③	3
Wang 2015 (Wang, 2015)	53/53	30:23/28:25	47.5/46	2HRZE/4HR scheme	BGD + C	90d	①	3
Wang 2014 (S. and W. 36 Cases of, 2014)	36/36	23:13/24:12	46.88 ± 3.98/47.52 ± 5.43	Conventional anti tuberculosis treatment with biomedicine	BGD + C	90d	①	3
Chen 2012 (Chen, 2012)	31/31	17:14/23:8	NA	3PaAmThVZ/15PaThVZ scheme	BGD + C	90d	②	2
Li 2019 (Li, 2019)	42/42	20:22/24:18	41.6 ± 0.5/40.5 ± 0.5	Conventional anti tuberculosis treatment with biomedicine	BGD + C	NA	①⑤⑦	2
Wang 2013 (Wang, 2013)	30/30	19:11/18:12	48.3 ± 18.2/47.9 ± 19.1	2HBZES/6HBE scheme	BGD + C	90d	①②③	5
Fan 2021 (Fan et al., 2021)	30/30	15:15/16:14	49.52 ± 3.26/49.50 ± 3.20	2HRZE/4HR scheme	BGD + C	180d	①③④	5
Han 2011 (Han et al., 2011)	40/40	25:15/19:21	66.5/64.5	Conventional anti tuberculosis treatment with biomedicine	BGD + C	14d is one course of treatment, with 4 courses of treatment	①②	3
Sun 2016 (Sun, 2016)	60/60	30:30/31:29	44.51 ± 12.8/46.25 ± 13.6	Initial treatment 2HRZE/4HR, re treatment 2HRZSE/5HRE	BGD + C	180–240d	①③④	3
Zhang 2022 (Zhang, 2022)	31/31	17:14/18:13	49.25 ± 6.56/49.30 ± 6.40	3HRZE/6HR scheme	BGD + C	60d is one course of treatment, with 3 courses of treatment	①⑤⑥⑦	5
Zhang 2023 (Zhang, 2023)	40/40	24:16/22:18	66.05 ± 7.26/65.93 ± 7.14	2HRZE/4HR scheme	BGD + C	90d	①⑤⑥⑦⑨	5
Zhang 2017 (Zhang, 2017)	50/50	26:24/23:27	45.3 ± 13.6/40.8 ± 13.6	2HRZE/4HR scheme	BGD + C	180d	①②③④	4
Zhao 2020 (Zhao and Wang, 2020)	41/41	24:17/22:19	46.73 ± 5.62/46.85 ± 5.17	2HRZE/4HR scheme	BGD + C	180d	①⑧	4
Wang 2020 (Wang et al., 2020a)	43/43	25:18/24:19	53.01 ± 4.45/52.49 ± 4.56	2HRZE/4HR scheme	BGD + C	180d	①③⑧	4
Ju 2019 (Ju, 2019)	21/21	12:9/11:10	44.5 ± 3.6/44.0 ± 3.5	biomedicine anti tuberculosis treatment (6Z-AM-V-PAS-Pto-Cs)	BGD + C	180d	①⑧	2
Zhong 2021 (Zhong et al., 2021)	46/46	24:22/26:20	39.1 ± 5.1/41.3 ± 5.3	2HRZE/4HR scheme	BGD + C	180d	①③⑤⑥	3
Yang 2016 (Yang et al., 2016)	30/30	15:15/16:14	48/50	HRZE quadruple therapy alone	BGD + C	7d is one course of treatment, with 1 month of treatment	①	3
Cheng 2020 (Cheng et al., 2020)	47/47	26:21/28:19	66.9 ± 1.5/67.1 ± 1.8	2HRZE/4HR scheme	BGD + C	180d	①③④⑧	5
Xiong 2019 (Xiong, 2019)	44/44	24:20/25:19	38.43 ± 2.43	Conventional anti tuberculosis treatment with biomedicinee	BGD + C	180d	①②③④	4
Zhou 2020 (Zhou, 2020)	47/47	23:24/26:21	36.11 ± 8.58/37.85 ± 9.61	Conventional anti tuberculosis treatment with biomedicine	BGD + C	180d	①⑤⑥⑦	5
Wang 2020 (Wang et al., 2020b)	36/36	19:17/21:15	48.3 ± 10.4/47.8 ± 11.6	The treatment plan formulated according to the “New Guidelines for the Treatment of Multidrug resistant tuberculosis” proposed by WHO	BGD + C	90d	①②③	3
Ma 2020 (Ma, 2020)	36/36	16:20/19:17	34.8 ± 4.6/38 ± 4.9	Conventional anti tuberculosis treatment with biomedicine	BGD + C	NA	②⑦	3

Am: Amikacin; BGD: baihe gujin decoction; C: control group; Cm: Capreomycin; Cs: Cycloserine; d: Days; E: ethambutol; H: isoniazid; Km: Kanamycin; Lfx (V): levofloxacin; Mfx: Moxifloxacin; NA: no relevant information; PAS: paza-aminosalicylate; Pto/Pa/Th: Protionamide; Qod: Take medication the next day; R: rifampicin; S: streptomycin; T: treatment group; Z: pyrazinamide.

①: the clinical efficacy rate ②: the lesions absorption rate ③: the sputum negative conversion rate ④: the changes in cavities ⑤: the incidence of totl adverse reactions ⑥: the incidence of liver function abnormalities ⑦: the incidence of gastrointestinal reactions ⑧: the CD4^+^ T lymphocyte subset levels.

### 3.3 Clinical efficacy rate

**FIGURE 4:**
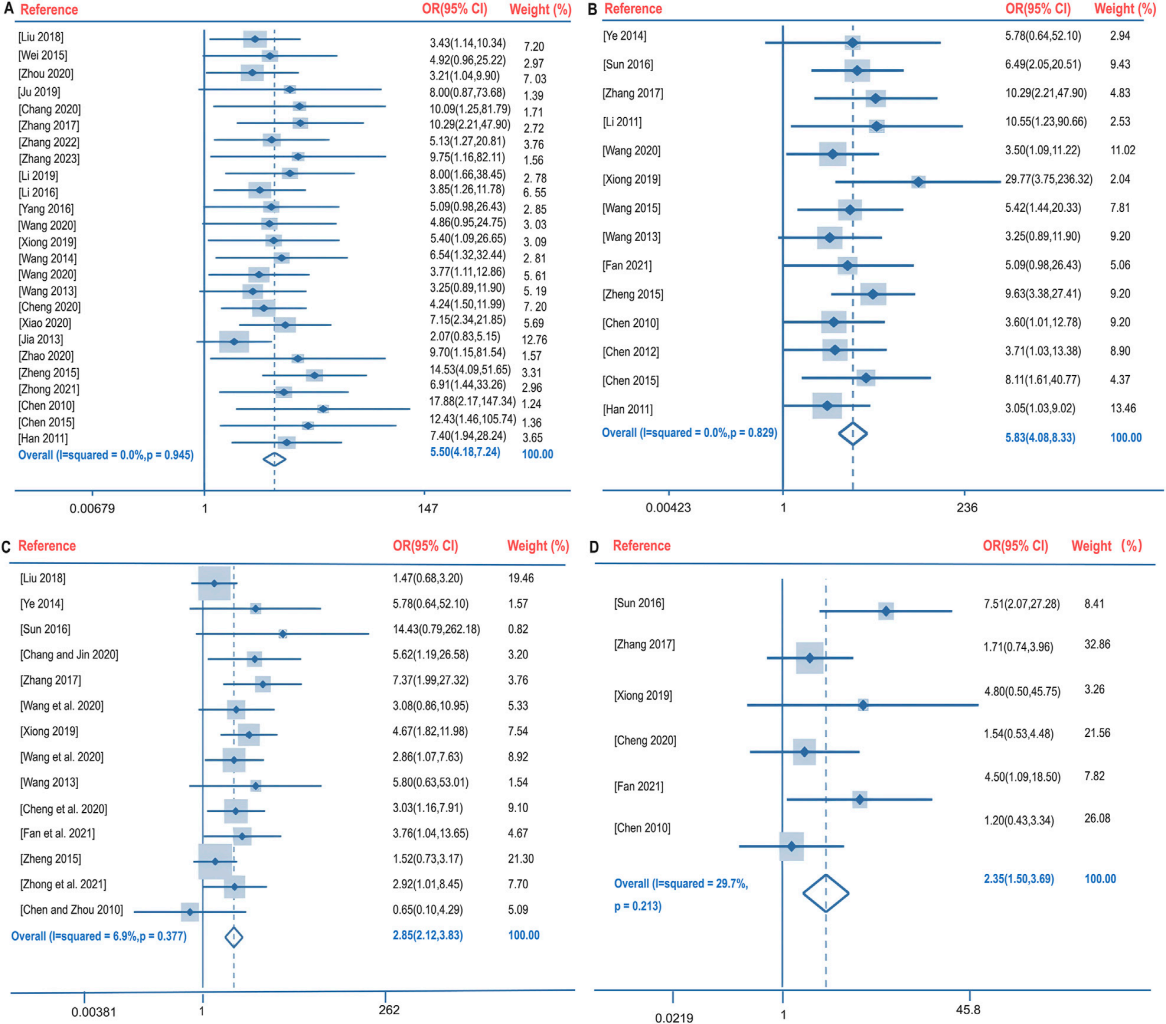
Forest plot of the clinical efficacy rate **(A)**, lesions absorption rate **(B)**, sputum negative conversion rate **(C)**, and changes in cavities **(D)**.

Clinical efficacy is defined as a significant improvement in the clinical symptoms of the patient after treatment, supported by imaging (e.g., chest X-ray or CT), and a comprehensive assessment in combination with laboratory indices. The clinical efficacy rate of BGD combined with chemotherapy for PTB treatment was compared with chemotherapy alone across 28 studies (Chang and Jin, 2020; Chen et al., 2015; Chen and Zhou, 2010; Cheng et al., 2020; Han et al., 2011; Jia, 2013; Ju, 2019; Li, 2019; Liu, 2018; Wang, 2013; Wang M. et al., 2020; Wang, 2014; Wang Y. et al., 2020; Wei, 2015; Xiao, 2020; Xiong, 2019; Yang et al., 2016; Zhang, 2017; Zhang, 2023; Zheng, 2015; Wang, 2015; Fan et al., 2021; Sun, 2016; Zhang, 2022; Zhao and Wang, 2020; Zhong et al., 2021; Zhou, 2020; Li, 2016). No significant heterogeneity was observed (*P* = 0.945, I^2^ = 0%), leading to the use of a fixed-effects model for meta-analysis. The results indicated a significantly higher clinical efficacy rate in the treatment group compared to the control group [OR = 5.50, 95%CI (4.18, 7.24), *P* < 0.05], as shown in Figure 4A.

### 3.4 Lesion absorption rate

Lesion absorption refers to the reduction in lesion size observed through imaging techniques. The lesion absorption rate following BGD combined with chemotherapy was compared with chemotherapy alone in 11 studies (Chen et al., 2015; Chen and Zhou, 2010; Han et al., 2011; Wang, 2013; Wang Y. et al., 2020; Xiong, 2019; Zhang, 2017; Zheng, 2015; Ye, 2014; Chen, 2012; Li, 2011). Again, no significant heterogeneity was found (*P* = 0.829, I^2^ = 0%), and a fixed-effects model was utilized. The treatment group demonstrated a significantly better lesion absorption rate than the control group [OR = 5.83, 95%CI (4.08, 8.33), *P* < 0.05], as illustrated in Figure 4B.

### 3.5 Sputum negative conversion rate

The sputum negative conversion rate after BGD combined with chemotherapy was compared with chemotherapy alone in 15 studies (Chang and Jin, 2020; Chen and Zhou, 2010; Cheng et al., 2020; Liu, 2018; Wang, 2013; Wang M. et al., 2020; Wang Y. et al., 2020; Xiong, 2019; Zhang, 2017; Zheng, 2015; Fan et al., 2021; Sun, 2016; Zhong et al., 2021; Ye, 2014; Ma, 2020). No significant heterogeneity was present (*P* = 0.377, I^2^ = 6.9%), and a fixed-effects model was applied. The treatment group had a higher sputum negative conversion rate than the control group, with a statistically significant difference [OR = 2.85, 95%CI (2.12, 3.83), *P* < 0.05], depicted in Figure 4C.

### 3.6 Changes in cavities

The changes in cavities following BGD combined with chemotherapy were compared with chemotherapy alone in six studies (Chen and Zhou, 2010; Cheng et al., 2020; Xiong, 2019; Zhang, 2017; Fan et al., 2021; Sun, 2016). No significant heterogeneity was detected (*P* = 0.213, I^2^ = 29.7%), and a fixed-effects model was employed. The treatment group showed significantly better cavity changes than the control group [OR = 2.35, 95%CI (1.50, 3.69), *P* < 0.05], as shown in Figure 4D.

### 3.7 Safety evaluation indicators

**FIGURE 5:**
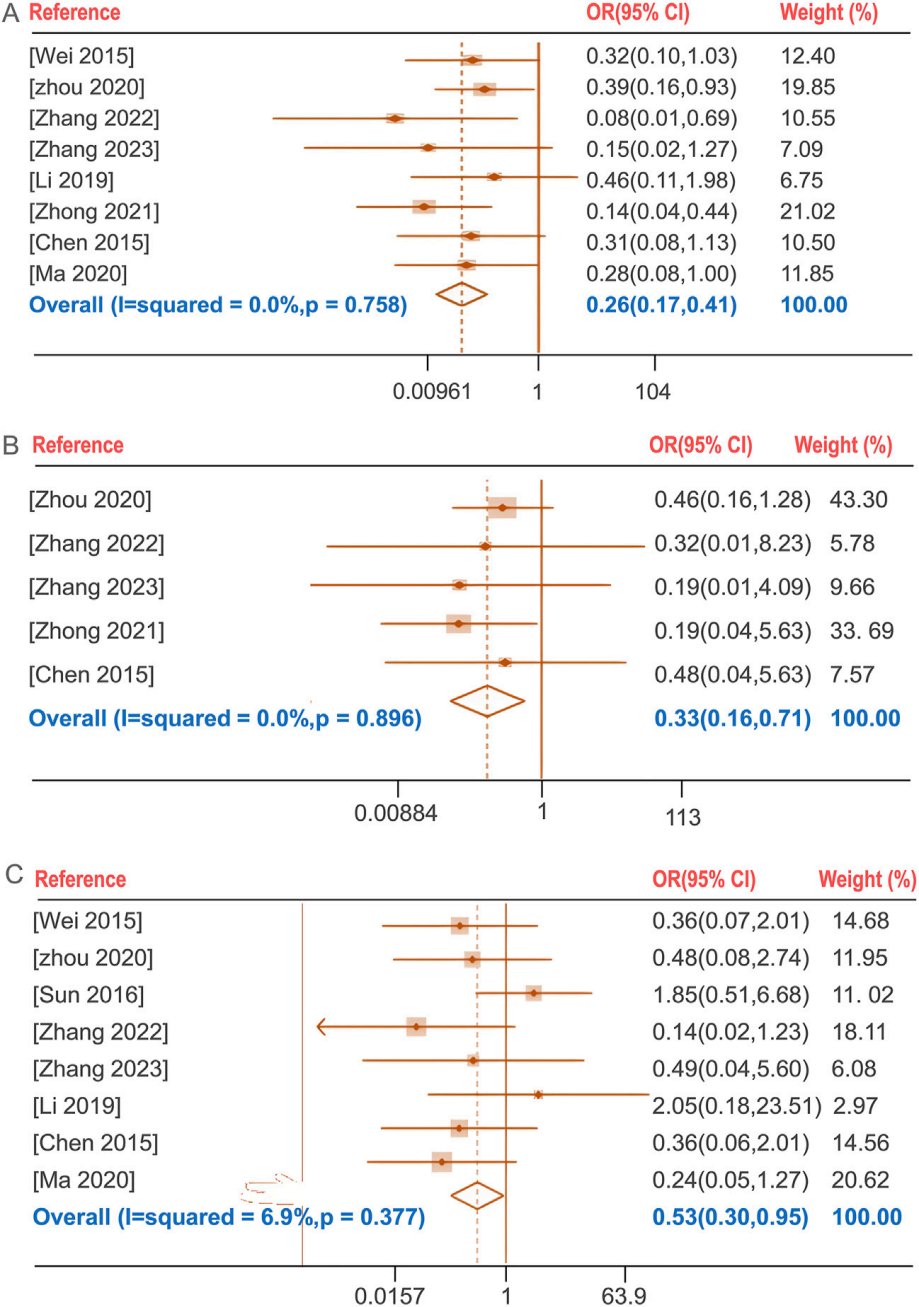
Forest plot of the incidence of total adverse reactions **(A)**, the incidence of liver function abnormalities **(B)**, the incidence of gastrointestinal reactions **(C)**.

The incidence of total adverse events following BGD combined with chemotherapy was compared with chemotherapy alone in seven studies (Chen et al., 2015; Li, 2019; Wei, 2015; Zhang, 2023; Zhang, 2022; Zhong et al., 2021; Zhou, 2020). No significant heterogeneity was found (*P* = 0.758, I^2^ = 0%), and a fixed-effects model was used. The treatment group exhibited a lower incidence of total adverse reactions compared to the control group [OR = 0.26, 95%CI (0.17, 0.41), *P* < 0.05]. The total adverse reactions include gastrointestinal reactions (nausea, vomiting, dyspepsia, diarrhea, *etc.*), allergic reactions (rash), Blood system (leukopenia), liver function abnormalities and neurological reactions (headache, dizziness, drowsiness, *etc.*). Notably, the incidence of liver function abnormalities and gastrointestinal reactions showed statistically significant differences with OR (95%CI) values of 0.33 (0.16, 0.71) and 0.53 (0.30, 0.95), respectively (P < 0.05) (Figure 5).

### 3.8 CD4^+^ T lymphocyte subset levels

**FIGURE 6:**
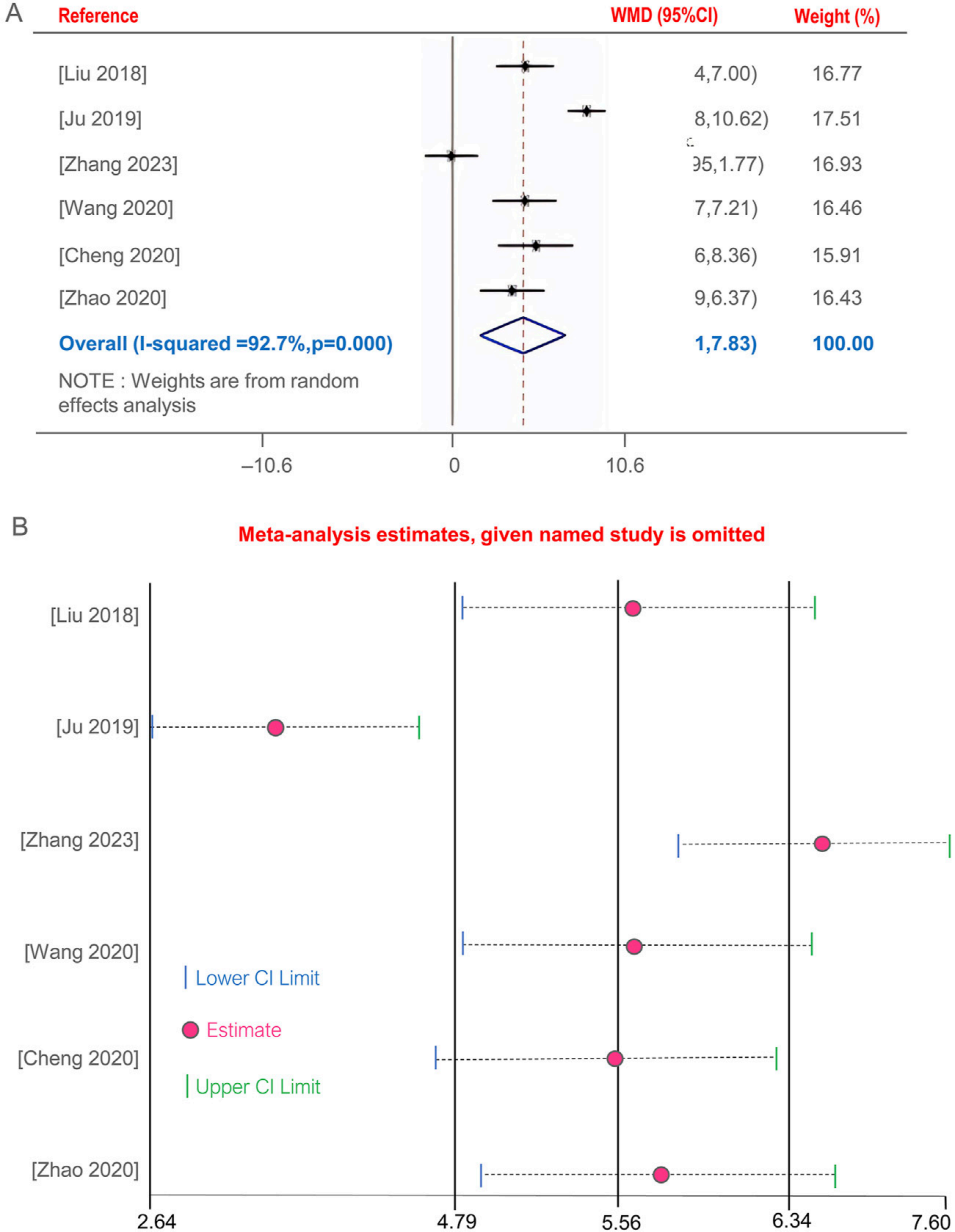
Forest plot of the CD4^+^ T lymphocyte subset levels **(A)**, Sensitivity analysis of the CD4^+^ T lymphocyte subset levels **(B)**.

Five studies (Cheng et al., 2020; Ju, 2019; Liu, 2018; Wang M. et al., 2020; Zhang, 2023; Zhao and Wang, 2020)evaluated the improvement of CD4^+^ T lymphocyte subset levels before and after BGD treatment for PTB. The combined therapy with BGD significantly enhanced immune status, with a weighted mean difference [OR = 4.87, 95%CI (1.91, 7.83), *P* < 0.05] (Figure 6A). Due to high heterogeneity was observed (*P* = 0, I^2^ = 92.7%). Sensitivity analysis through one-by-one exclusion did not reveal a cause for the high heterogeneity, and the overall results remained statistically significant, indicating robust findings, as shown in Figure 6B.

### 3.9 Publication bias

**FIGURE 7:**
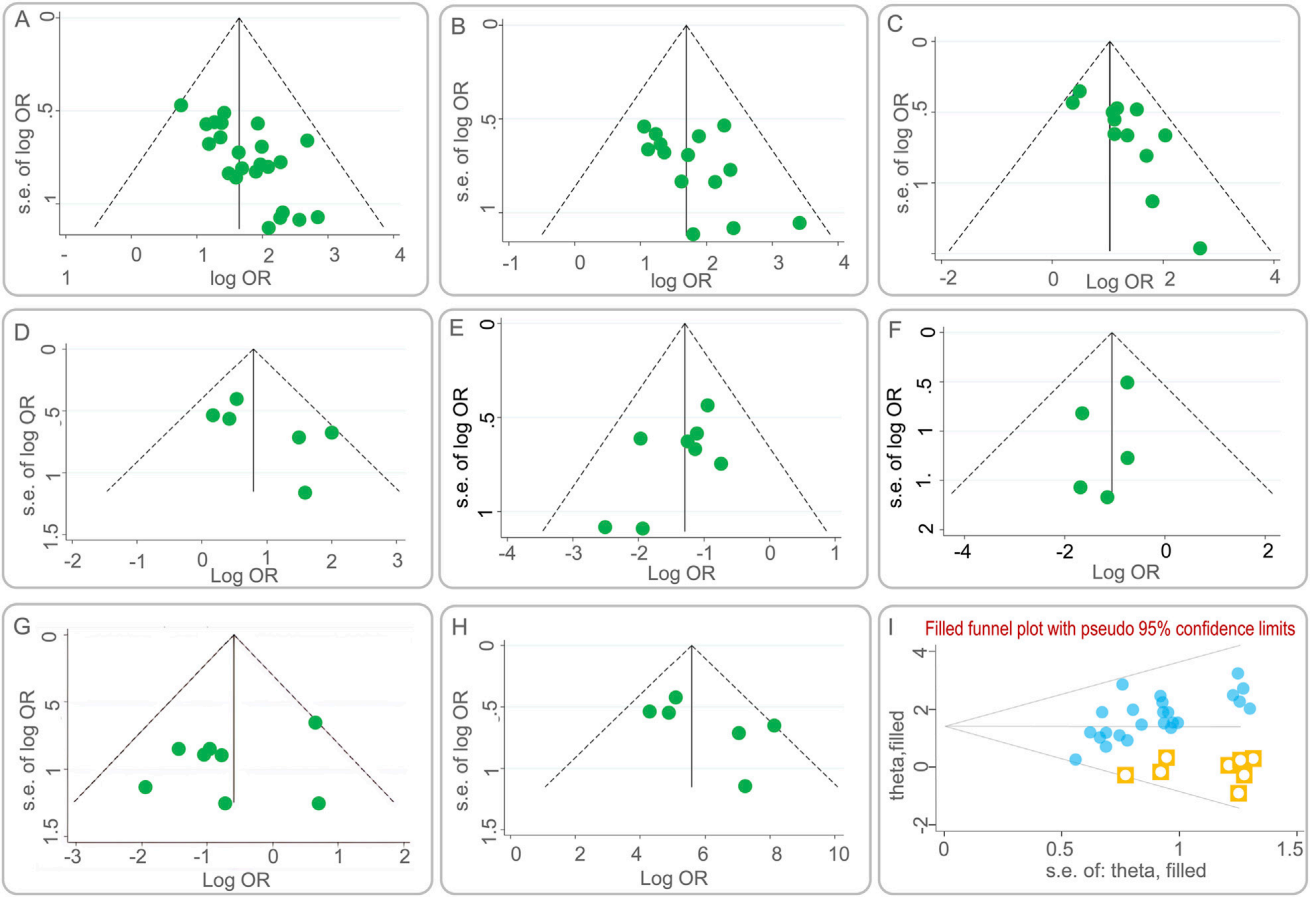
Funnel plots of publication bias. **(A)** the clinical efficacy rate; **(B)** the lesion absorption rate; **(C)** the sputum negative conversion rate; **(D)** the changes in cavities; **(E)** the incidence of total adverse reactions; **(F)** the incidence of liver function abnormalities; **(G)** the incidence of gastrointestinal reactions; **(H)** the CD4^+^ T lymphocyte subset levels; **(I)** Funnel plot of the clinical efficacy rate after using the trim-and-fill method.

Publication bias was assessed using funnel plots, a graphical representation that can indicate the presence of asymmetry, suggesting potential bias. Figures 7A–H presents the funnel plots for various outcome measures. Symmetrical funnel plots for the lesion absorption rate and the incidence of liver function abnormalities suggest that the combination of BGD with chemical drugs is more effective. However, asymmetry in the funnel plots for the clinical efficacy rate, sputum negative conversion rate, changes in cavities, incidence of total adverse reactions, incidence of gastrointestinal reactions, and CD4^+^ T lymphocyte subset levels indicates potential publication bias.

To quantitatively assess publication bias, Egger’s test was employed using Stata 15.1. The results are detailed in Table 3. While the P-values for most outcome measures were greater than 0.05, indicating no significant publication bias, the clinical efficacy rate showed a P-value less than 0.05, suggesting the presence of publication bias.

**TABLE 3:** Egger’s test for publication bias in outcome indicators after Baihe Gujin Decoction combined with anti-tuberculosis drugs for the treatment of pulmonary tuberculosis.

Index	P value
The clinical efficacy rate	0.029
The lesion absorption rate	0.997
The sputum negative conversion rate	0.668
The changes in cavities	0.279
The incidence of total adverse reactions	0.294
The incidence of liver function abnormalities	0.495
The incidence of gastrointestinal reactions	0.141
The CD4^+^ T lymphocyte subset levels	0.510

To further evaluate the impact of publication bias on the clinical efficacy rate, a trim and fill analysis was conducted. Figure 7I illustrates the results after adjusting for potential missing studies that could cause asymmetry. The inclusion of data from eight hypothetical studies, derived from the trim and fill method, was used to re-estimate the effect size. The adjusted results showed a heterogeneity test Q-value of 26.672, with a log OR of 1.434 and a 95% CI of (1.180, 1.688). This suggests that the initial effect size estimation was minimally affected by publication bias, reinforcing the robustness of the study’s findings.

## 4 Evaluation of the quality of evidence

The quality of evidence for each outcome indicator was assessed using the GRADE pro GDT system, with RCTs without major flaws preset as the highest level of evidence in GRADE. Evidence was evaluated for quality and processed according to five downgrading factors. The results suggested that the quality of evidence was moderate in Changes in Cavities and low quality for the remaining indicators (As shown in Table 4).

**TABLE 4:** GRADE evidence quality evaluation form.

Outcome indicator	Limitations of the study	Incoherence	Indirect	Inaccuracy	Publish bias	Sample size (C/T)	Quantity of effect (95%*CI*)	Quality of evidence
Clinical Efficacy Rate	Insignificant^①^	Insignificant	Insignificant	Insignificant	Undetected	1,242/1,250	OR = 5.50 (4.18, 7.24)	Low quality
Lesion Absorption Rate	Insignificant^①^	Insignificant	Insignificant	Insignificant	Non-serious	393/399	OR = 5.83 (4.08, 8.33)	Low quality
Sputum Negative Conversion Rate	Insignificant^①^	Insignificant	Insignificant	Insignificant	Serious	683/689	OR = 2.85 (2.12, 3.83)	Low quality
Changes in Cavities	Insignificant^①^	Insignificant	Insignificant	Insignificant	Serious	266/271	OR = 2.35 (1.50, 3.69)	Moderate quality
the incidence of liver function abnormalities	Insignificant^①^	Insignificant	Insignificant	Serious^③^	Non-serious	194/194	OR = 0.33 (0.16, 0.71)	Low quality
the incidence of gastrointestinal reactions	Insignificant^①^	Insignificant	Insignificant	Insignificant	Serious	260/260	OR = 0.53 (0.30, 0.95)	Low quality
CD4^+^ T Lymphocyte Subset Levels	Insignificant^①^	Serious^②^	Insignificant	Insignificant	Serious	206/206	OR = 4.87 (1.91, 7.83)	Low quality

C: control group; T: treatment group; *CI*: confidence interval; OR: odds ratio.

①:Most of the literature does not mention blinding and some of the literature does not mention allocation concealment methods, thus reducing the level of evidence due to risk of bias; ②:*I* ^2^ > 50%; ③: The study involved a sample size of <400 individuals.

## 5 Discussion

PTB is characterized by symptoms such as cough, hemoptysis, fever, night sweats, and weight loss. Despite advances in TB management, it remains a significant global health challenge, with high incidence and mortality rates (Zhuang et al., 2020; Gong et al., 2024; Gong and Du, 2024a). The effectiveness of current chemotherapy treatments is increasingly compromised by the emergence of multidrug-resistant TB strains. TCM, with its long-standing understanding of TB, emphasizes the importance of lung health and the potential spread to other organs. The TCM approach to treating TB focuses on strengthening the lungs, eradicating the disease, and eliminating pathogens. There is a growing body of evidence highlighting the therapeutic benefits of BGD in TB treatment, advocating for its broader clinical application (Pan et al., 2022; Yang et al., 2022; Yuan and Zhao, 2018).

BGD, a renowned anti-TB formulation derived from “Shenzhai Yishu,” is frequently utilized as an adjunct to conventional TB treatment. It is composed of *Lilium Lancifolium* Thunb, *R. glutinosa* (Gaertn.) DC., Angelica sinensis (Oliv.) Diels, *P. lactiflora Pall.*, *G. uralensis Fisch. ex DC.*, *P. grandiflorus* (Jacq.) A.DC., *S. ningpoensis* Hemsl, *F. cirrhosa* D. Don, and *O. japonicus* (Thunb.) Ker Gawl. It is favored for its capacity to enhance clinical efficacy and modulate immunological responses. Modern pharmacological studies have revealed that BGD contains various bioactive metabolites, including saponins, polysaccharides, phenolic glycerides, flavonoids, and alkaloids (Zhou J. et al., 2021). For example, *Lilium Lancifolium Thunb*. has been shown to possess anti-inflammatory effects by modulating specific inflammatory pathways (Kwon et al., 2010). Kwon et al. found that anti-inflammatory effects of methanol extracts of the root of *Lilium Lancifolium Thunb.* are due to downregulation of iNOS and COX-2 *via* suppression of NF-κB activation and nuclear translocation as well as blocking of ERK and JNK signaling in LPS (Lipopolysaccharide) stimulated Raw264.7 cells (Kwon et al., 2010). Some studies have found that polysaccharides extracted from *Lilium Lancifolium Thunb.* have immune-enhancing effects and can achieve immune enhancement by increasing monocyte macrophage phagocytic activity, thymic and splenic indices, serum-specific antibody capacity, and proliferation of splenocytes in mice (Pan et al., 2017; Mi et al., 2007). Among them, the thymic and splenic indices reflect the state of the immune system in experimental animals, and changes in their weights may be associated with changes in immune function (Calculation Formula: thymic index = Thymus Weight (mg)/Animal Body Weight (g); splenic index = Spleen Weight (mg)/Animal Body Weight (g)). The polysaccharide extracted from *R. glutinosa* (Gaertn.) DC can enhance cellular immune function. Zhou et al. found that two polysaccharides (SDH-WA and SDH-0.2A) extracted from *R. glutinosa* (Gaertn.) DC showed cytotoxic effects on RAW264.7 cells, significantly promoted their phagocytic activity and induced the production of TNF-α and IL-6 in RAW264.7 cells (Zhou Y. et al., 2021). The extracted from Angelica sinensis (Oliv.) Diels exert an anti-inflammatory effect on LPS-induced RAW 264.7 *via* NO-bursting/calcium-mediated JAK-STAT pathway (Kim et al., 2018). *Paeonia lactiflora Pall*. has stronger antioxidant activity (Sun et al., 2022), in addition, its extracts are effective in alleviating mucus producing respiratory inflammation, through inhibits the production of MUC5AC mucin protein and gene expression in TNF-α-induced H292 cells and may be closely related to the modulation of the activation of the ERK signaling pathway (Han et al., 2023). *Glycyrrhiza uralensis Fisch. ex DC*. may exert anti-inflammatory effects by inhibiting the release of pro-inflammatory mediators and cytokines and down-regulating the expression of iNOS and COX-2 mRNA (Jiang et al., 2021). It has been found that the extracted from *S. ningpoensis* Hemsl. may exert anti-inflammatory effects by modulating the NF-κB signaling pathway (Shin et al., 2020). *Fritillaria cirrhosa* D. Don may exert anti-inflammatory effects through various pathways. It has been found that the extracted from *F. cirrhosa* D. Don can mitigate pulmonary structural impairment and inhibit inflammatory responses by mediating expression of cytokines, such as IL-1β, IL-6, IL-8, TNF-α, NF-κB, TGF-β1, MMP-9, and TIMP-1 (Wang et al., 2016). *Ophiopogon japonicus* (Thunb.) Ker Gawl. is a Chinese herb with immunomodulatory effects, and malt polysaccharide is a significant member of its composition. Zhou et al. found that the polysaccharide extracted from *O. japonicus* (Thunb.) Ker Gawl. could attenuate macrophage damage induced by LPS, reduce inflammatory factors IL-1β, IL-6, IL-8, and TNF-α, inhibit the activation of NF-κB signaling pathway, reduce oxidative stress, and decrease the secretion of inflammatory factors (Zhou et al., 2019). In conclusion, BGD can effectively assist in treating tuberculosis thanks to the synergistic effect of multiple botanical drugs. Polysaccharides from *P. grandiflorus* (Jacq.) A.DC. can increase the proliferative activity of lymphocytes, promote the progression of the lymphocyte cycle from the G0/G1 phase to the S and G2/M phases and, and simultaneously, increase the levels of CD4^+^ and CD8^+^ T cells (Zhao et al., 2017). Specifically speaking, when MTB infects the human body, it triggers both innate and adaptive immune responses, and CD4^+^ T cells, a subset of T lymphocytes, are crucial in TB immunomodulation (Zhuang et al., 2023; Li et al., 2023; Jiang et al., 2023). In previous studies, mice or human lacking CD4^+^ T cells are more susceptible to TB, highlighting their key role in the disease’s immunology (Leveton et al., 1989; Gong and Du, 2024b; Gong and Du, 2023; Gong et al., 2023; Yang et al., 2023). The findings of this meta-analysis indicate that BGD, in combination with anti-TB Chemotherapy, can significantly increase CD4^+^ T lymphocyte subset levels, suggesting an immunomodulatory effect (Yang et al., 2021; Xie et al., 2023). Meanwhile, specific botanical drug metabolites can regulate the immune function, thereby improving the clinical symptoms and accelerating the recovery process of TB patients. However, whether there is a direct link between these mechanisms of action still needs to be demonstrated in further studies.

Although biomedicine has significant efficacy in treating pulmonary tuberculosis, which is currently our clinical preference, the inevitable adverse reactions remain. Combining TCM with biomedicine to assist in the treatment of pulmonary tuberculosis has achieved integration of Chinese and biomedicine, complementing each other’s strengths and weaknesses, accelerating the improvement of patients’ conditions, thereby improving the treatment efficacy and reducing the incidence of adverse reactions. In addition to Baihe Gujin Decoction, Chinese patent medicine for treating of pulmonary tuberculosis also includes various Chinese medicines, such as JieHeWan, Shen Ling Bai Zhu San, Astragalus Injection, and TanReqing Injection, from the increase in clinical effectiveness, reduction in sputum culture conversion rate and incidence of adverse reactions, it can be seen that the effectiveness of treating pulmonary tuberculosis with the combination of Chinese medicine and anti-tuberculosis drugs is better than using anti-tuberculosis drugs alone, and is safer (Yan and Gao, 2017; Ding et al., 2017). They play a complementary role to a certain extent, possibly by inhibiting inflammatory cytokines to reduce pulmonary inflammation, enhance immune function, and accelerate recovery. However, given the complexity and individual differences of Chinese medicine, it is particularly important to use it reasonably and safely. Botanical drug decoctions are not like fixed commercial Chinese polyherbal preparations (CCPP), they can be adjusted flexibly according to the patient’s condition, controlling the intake amount from the source to further reduce the incidence of adverse reactions in patients. In addition to the therapeutic effect, the potential interaction of BGD with traditional anti-TB Chemotherapy is also of concern. Numerous studies have demonstrated that the combination of the two treatments can more effectively reduce inflammatory factor levels in patients, enhance their immune function, and result in no significant adverse reactions (Guo, 2014; Liu et al., 2023; Li et al., 2024).

This meta-analysis systematically reviewed 32 RCTs, showing that BGD combined with anti-TB Chemotherapy is significantly more effective and safer than conventional chemotherapy alone. The findings are multifaceted: (1) A rigorous literature search was conducted across seven databases, adhering to PRISMA guidelines and yielding studies with a moderate Jadad score; (2) Various outcome measures revealed the efficacy and safety advantages of BGD combination therapy for PTB; (3) The combination therapy significantly reduced the incidence of total adverse reactions; (4) The therapy also significantly increased CD4^+^ T lymphocyte subset levels; (5) High heterogeneity and publication bias were addressed through sensitivity analysis and statistical tests, confirming the robustness of the results.

However, this study has several limitations that warrant consideration. First, the methodological quality of included RCTs exhibited significant heterogeneity, with common issues including limited sample sizes, inconsistent treatment protocols across studies, and insufficient reporting of randomization procedures. Second, substantial variations in BGD dosage regimens (e.g., decoction concentration, administration frequency) and observation periods were noted, compounded by the absence of standardized efficacy evaluation criteria. Third, critical phytochemical parameters—such as the mass ratios of constituent herbs and extraction methodologies—were frequently omitted in original studies, potentially undermining the pharmacological reproducibility and cross-trial comparability. Furthermore, methodological shortcomings (e.g., lack of blinding and allocation concealment) may have introduced selection and performance biases.

To address these limitations, future investigations should prioritize large-scale, multicenter, randomized, double-blind, placebo-controlled trials with rigorous allocation concealment. Concurrently, establishing standardized quality control protocols for BGD preparation and harmonized clinical endpoints is imperative. As mechanistic studies continue to elucidate BGD’s multi-target pharmacological actions—particularly its immunomodulatory and organ-protective properties—this formulation holds promising potential for expanded therapeutic applications in both disease prevention and integrated management strategies.

## 6 Conclusion

This meta-analysis, encompassing 32 RCTs, demonstrates that BGD can enhance clinical efficacy, facilitate lesion regression, and mitigate the side effects of pharmaceutical drugs, underscoring its therapeutic potential as an adjuvant treatment for PTB. The results indicate that BGD may offer significant benefits in the management of PTB, contributing to a more effective and safer treatment strategy. However, the quality of evidence, as indicated by the Jadad score, varies, with many studies falling into the moderate or low quality categories. This variability underscores the need for future research to be conducted with greater methodological rigor. Large-scale, randomized, double-blind, high-quality trials are essential to further substantiate the efficacy and safety of BGD in the treatment of PTB.

As we look to the future, it is anticipated that an increasing number of high-quality RCTs will be conducted on a global scale. With these studies, the mechanisms of action of BGD *in vivo* will be elucidated with greater clarity. This enhanced understanding will not only bolster the evidence base for BGD’s role in PTB treatment but also expand its application in the broader spectrum of disease prevention and management.

## Data Availability

All data generated or analyzed during this study were included in this published article and its supplementary information files.

## Abbreviations

BGD, Baihe Gujin Decoction; LPS, lipopolysaccharide; MTB, *M. tuberculosis*; ORs, Odds ratios; PTB, pulmonary tuberculosis; RCTs, randomized controlled trials; TB, tuberculosis; TCM, traditional Chinese medicine; WMDs, weighted mean differences; commercial Chinese polyherbal preparations (CCPP).
