# Supplementary material for: Efficacy and safety of baihe gujin decoction as an adjunct to chemotherapy in pulmonary tuberculosis: A systematic review and meta-analysis
Source: Front Pharmacol. 2025 May 13;16:1538692. doi: 10.3389/fphar.2025.1538692 (PMC12106383; doi:10.3389/fphar.2025.1538692)
Supplement: Supplementary file 1 [file Table1.docx]

**PRISMA 2020 Checklist**

**Location**

**where item**

**is reported**

**Section and**

**Topic**

**Item**

**#**

**Checklist item**

**TITLE**

Title

1

2

Identify the report as a systematic review.

**ABSTRACT**

Abstract

See the PRISMA 2020 for Abstracts checklist.

**INTRODUCTION**

Rationale

3

4

Describe the rationale for the review in the context of existing knowledge.

Objectives

Provide an explicit statement of the objective(s) or question(s) the review addresses.

**METHODS**

Eligibility criteria

5

6

Specify the inclusion and exclusion criteria for the review and how studies were grouped for the syntheses.

Information

sources

Specify all databases, registers, websites, organisations, reference lists and other sources searched or consulted to identify studies. Specify the

date when each source was last searched or consulted.

Search strategy

7

8

Present the full search strategies for all databases, registers and websites, including any filters and limits used.

Selection process

Specify the methods used to decide whether a study met the inclusion criteria of the review, including how many reviewers screened each record

and each report retrieved, whether they worked independently, and if applicable, details of automation tools used in the process.

Data collection

process

9

Specify the methods used to collect data from reports, including how many reviewers collected data from each report, whether they worked

independently, any processes for obtaining or confirming data from study investigators, and if applicable, details of automation tools used in the

process.

Data items

10a List and define all outcomes for which data were sought. Specify whether all results that were compatible with each outcome domain in each

study were sought (e.g. for all measures, time points, analyses), and if not, the methods used to decide which results to collect.

10b List and define all other variables for which data were sought (e.g. participant and intervention characteristics, funding sources). Describe any

assumptions made about any missing or unclear information.

Study risk of bias

assessment

11 Specify the methods used to assess risk of bias in the included studies, including details of the tool(s) used, how many reviewers assessed each

study and whether they worked independently, and if applicable, details of automation tools used in the process.

Effect measures

12 Specify for each outcome the effect measure(s) (e.g. risk ratio, mean difference) used in the synthesis or presentation of results.

Synthesis

methods

13a Describe the processes used to decide which studies were eligible for each synthesis (e.g. tabulating the study intervention characteristics and

comparing against the planned groups for each synthesis (item #5)).

13b Describe any methods required to prepare the data for presentation or synthesis, such as handling of missing summary statistics, or data

conversions.

13c Describe any methods used to tabulate or visually display results of individual studies and syntheses.

13d Describe any methods used to synthesize results and provide a rationale for the choice(s). If meta-analysis was performed, describe the

model(s), method(s) to identify the presence and extent of statistical heterogeneity, and software package(s) used.

13e Describe any methods used to explore possible causes of heterogeneity among study results (e.g. subgroup analysis, meta-regression).

13f Describe any sensitivity analyses conducted to assess robustness of the synthesized results.

Reporting bias

assessment

14 Describe any methods used to assess risk of bias due to missing results in a synthesis (arising from reporting biases).

Certainty

15 Describe any methods used to assess certainty (or confidence) in the body of evidence for an outcome.

assessment

**RESULTS**


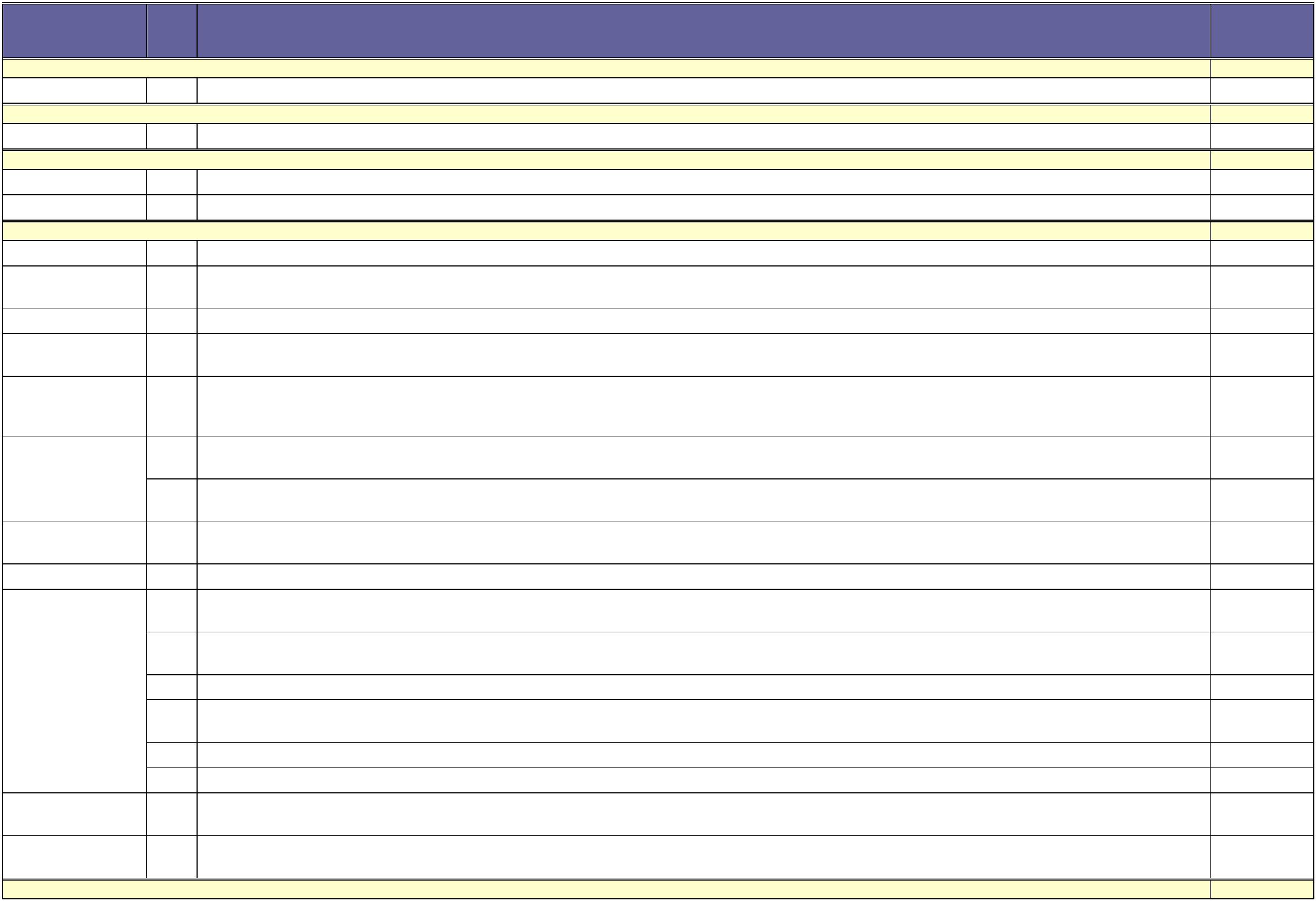

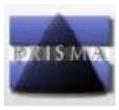


Methods

Methods

Methods

Methods

Methods

Methods

Abstract

Methods

Methods

Methods

Methods

Methods

Methods

Methods

Methods

Introduction

Introduction

Methods

Methods

Abstract

Title

**PRISMA 2020 Checklist**

**Location**

**where item**

**is reported**

**Section and**

**Topic**

**Item**

**#**

**Checklist item**

Study selection

16a Describe the results of the search and selection process, from the number of records identified in the search to the number of studies included in

the review, ideally using a flow diagram.

16b Cite studies that might appear to meet the inclusion criteria, but which were excluded, and explain why they were excluded.

17 Cite each included study and present its characteristics.

Study

characteristics

Risk of bias in

studies

18 Present assessments of risk of bias for each included study.

Results of

individual studies

19 For all outcomes, present, for each study: (a) summary statistics for each group (where appropriate) and (b) an effect estimate and its precision

(e.g. confidence/credible interval), ideally using structured tables or plots.

Results of

syntheses

20a For each synthesis, briefly summarise the characteristics and risk of bias among contributing studies.

20b Present results of all statistical syntheses conducted. If meta-analysis was done, present for each the summary estimate and its precision (e.g.

confidence/credible interval) and measures of statistical heterogeneity. If comparing groups, describe the direction of the effect.

20c Present results of all investigations of possible causes of heterogeneity among study results.

20d Present results of all sensitivity analyses conducted to assess the robustness of the synthesized results.

21 Present assessments of risk of bias due to missing results (arising from reporting biases) for each synthesis assessed.

22 Present assessments of certainty (or confidence) in the body of evidence for each outcome assessed.

Reporting biases

Certainty of

evidence

**DISCUSSION**

Discussion

23a Provide a general interpretation of the results in the context of other evidence.

23b Discuss any limitations of the evidence included in the review.

23c Discuss any limitations of the review processes used.

23d Discuss implications of the results for practice, policy, and future research.

**OTHER INFORMATION**

Registration and

protocol

24a Provide registration information for the review, including register name and registration number, or state that the review was not registered.

24b Indicate where the review protocol can be accessed, or state that a protocol was not prepared.

24c Describe and explain any amendments to information provided at registration or in the protocol.

25 Describe sources of financial or non-financial support for the review, and the role of the funders or sponsors in the review.

26 Declare any competing interests of review authors.

Support

Competing

interests

Availability of

data, code and

other materials

27 Report which of the following are publicly available and where they can be found: template data collection forms; data extracted from included

studies; data used for all analyses; analytic code; any other materials used in the review.

*From:* Page MJ, McKenzie JE, Bossuyt PM, Boutron I, Hoffmann TC, Mulrow CD, et al. The PRISMA 2020 statement: an updated guideline for reporting systematic reviews. BMJ 2021;372:n71. doi:

10.1136/bmj.n71

For more information, visit: <http://www.prisma-statement.org/>


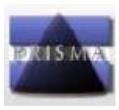

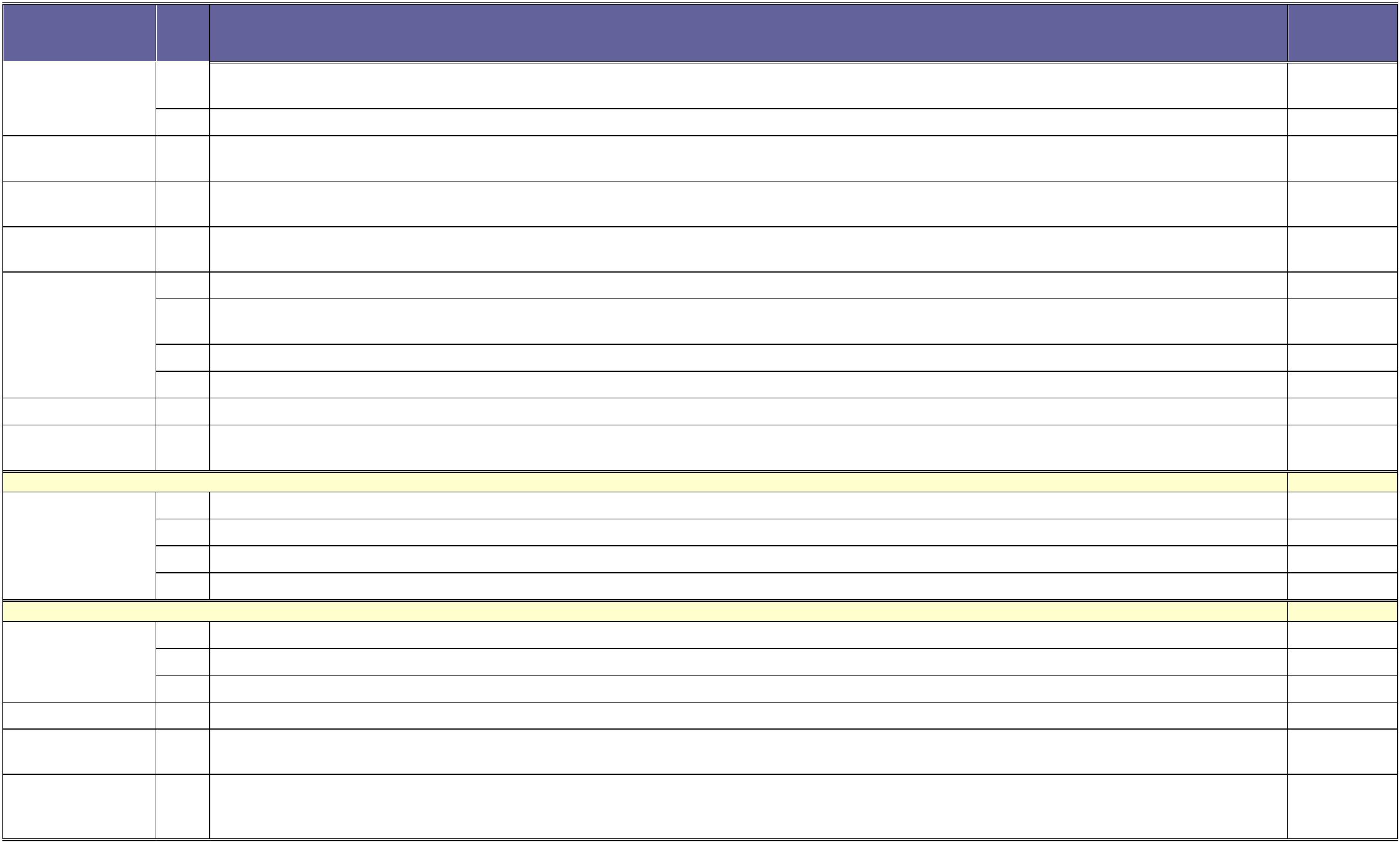

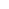


Discussion

Discussion

Discussion

Discussion

Discussion

Discussion

Methods

Results

NA

Methods

Methods

Results

Results

Results

Results

Results

Results

Results

Results

Results

Results
