# Supplementary material for: Efficacy and safety of baihe gujin decoction as an adjunct to chemotherapy in pulmonary tuberculosis: A systematic review and meta-analysis
Source: Front Pharmacol. 2025 May 13;16:1538692. doi: 10.3389/fphar.2025.1538692 (PMC12106383; doi:10.3389/fphar.2025.1538692)
Supplement: Supplementary file 2 [file Table2.docx]

Supplementary Material-Table S2-Jadad scale.

| Generation of randomization sequence | Inappropriate: use of alternate allocation e.g. single and double numbers  Unclear: randomized trial but no description of the method of random allocation  Appropriate: computer-generated random numbers or similar methods | 2  1  0 |
| --- | --- | --- |
| Randomization Hide | Unclear: indicates only the use of random number tables or other random allocation schemes  Unused  Inappropriate: Alternate assignments, case numbers, Sunday numbers, open random number tables, series coded envelopes, and any measure that does not prevent predictability of grouping  Appropriate: centre- or pharmacy- controlled dispensing protocols, or containers with consistent serial numbers, on-site computer control, sealed opaque envelopes, or other methods that make the dispensing sequence unpredictable to clinicians and subjects | 0  0  1  2 |
| Blinding | Inappropriate: failure to use double-blind or blinded methods are inappropriate, e.g., comparison of tablets and injections  Unclear: the test statement was blinded, but the method was not described  Appropriate: exactly the same placebo tablets or similar methods were used | 2  1  0 |
| Withdrawal and exit | No description of the number or reasons for withdrawal or exit  Describes the number of and reasons for withdrawals or exits | 1  0 |

Note: This table does not emphasize the quality factor of concealment of randomization.
